# The Influence of Cell Cycle Regulation on Chemotherapy

**DOI:** 10.3390/ijms22136923

**Published:** 2021-06-28

**Authors:** Ying Sun, Yang Liu, Xiaoli Ma, Hao Hu

**Affiliations:** 1 Institute of Biomedical Materials and Engineering, College of Materials Science and Engineering, Qingdao University, Qingdao 266071, China; sunying150996@163.com (Y.S.); ly150726429@163.com (Y.L.); 2 Qingdao Institute of Measurement Technology, Qingdao 266000, China; maxiaoli1989@yeah.net

**Keywords:** chemotherapy, cell cycle regulation, drug delivery systems, combination chemotherapy, cancer therapy

## Abstract

Cell cycle regulation is orchestrated by a complex network of interactions between proteins, enzymes, cytokines, and cell cycle signaling pathways, and is vital for cell proliferation, growth, and repair. The occurrence, development, and metastasis of tumors are closely related to the cell cycle. Cell cycle regulation can be synergistic with chemotherapy in two aspects: inhibition or promotion. The sensitivity of tumor cells to chemotherapeutic drugs can be improved with the cooperation of cell cycle regulation strategies. This review presented the mechanism of the commonly used chemotherapeutic drugs and the effect of the cell cycle on tumorigenesis and development, and the interaction between chemotherapy and cell cycle regulation in cancer treatment was briefly introduced. The current collaborative strategies of chemotherapy and cell cycle regulation are discussed in detail. Finally, we outline the challenges and perspectives about the improvement of combination strategies for cancer therapy.

## 1. Introduction

Chemotherapy is currently one of the main methods of tumor treatment [1]. However, there are still several obstacles to achieve the desired therapeutic effect. Some inevitable side effects such as nausea and hair loss usually accompany chemotherapy. In the course of chemotherapy, the multidrug resistance (MDR) of tumor cells will also affect the treatment. As a single chemotherapeutic drug usually cannot meet the requirements of clinical treatment, the combination of different chemotherapeutic drugs or treatments (e.g., chemotherapy and radiotherapy) is operated to prevent the replication, invasion, and metastasis of cancer cells [2]. These strategies are of great significance for reducing side effects, overcoming MDR, reducing the dosage of each drug, and finally improving the therapeutic effect. For example, the combination of hydrophilic gemcitabine (GEM) and hydrophobic paclitaxel (TAX) shows synergistic activity and non-overlapping toxicity [3]. This synergistic strategy has been proven to effectively inhibit the proliferation of cancer cells. Clinically, vincristine (VCR) can be used to block tumor cells in the M phase. After 6 to 8 h, the pancreatic cancer cells will enter the G1 phase synchronously, and then cyclophosphamide (CTX) can be operated to kill tumor cells in the G1 phase effectively.

Cell cycle regulation has an important influence on the proliferation, metastasis, and recurrence of tumor cells [4]. In the current cancer treatment, the regulation of the cell cycle is mainly to control the expression of related genes and the activity of intracellular enzymes, proteins, or signal factors [5,6,7]. For example, chemotherapeutic drugs such as methotrexate (MTX) or 5-fluorouracil (5-FU) mainly destroy the synthesis of nucleotides and DNA replication in the process of cell proliferation [8,9]. Curcumin can regulate growth factors, enzymes, transcription factors, kinases, inflammatory cytokines, and proapoptotic (by upregulation) and antiapoptotic (by downregulation) proteins [10]. In addition, curcumin can also regulate the growth of tumor cells by regulating a variety of cell signaling pathways, and enhance the effect of chemotherapeutic drugs and radiation on cancer [11]. Tyrosine kinase inhibitors (TKIs) can reverse MDR by blocking the function of ABC transporters, thus promoting drug accumulation and enhancing the efficacy of conventional chemotherapy [12]. Some chemotherapeutic drugs can inhibit cell cycle progression, such as 3,3′, 5,5′-tetramethoxydiphenyl-4,4′-diol (TMBP) and Asparinin A [13,14]. Similarly, some chemotherapeutic drugs can promote cell cycle progression, such as tyrosine kinase inhibitors MK-5108 and LY2603618 [15,16]. Chemotherapeutic drugs can also be divided into cell cycle nonspecific agents (CCNSA) and cell cycle specific agents (CCSA) according to their effects on cell cycle [17]. For example, alkylating agents and anthracyclines belong to CCNSA, which can act on all stages of the proliferating cell population, including cells in G0 phase [18]. CCSA mainly affects a certain phase of the cell cycle [19]. For example, 6-mercaptopurine (6-MP) as an antimetabolic drug mainly plays a role in the S phase while plant alkaloids mainly act on M-phase cells [20,21].

In this review, we first briefly introduce the mechanism of action of commonly used chemotherapeutic drugs and the effect of cell cycle on tumorigenesis and development, and then discuss the collaborative strategies of cell cycle regulation and chemotherapy. We also summarize the exemplary research works on the collaborative strategies for tumor therapy.

## 2. Mechanism of Chemotherapeutic Drugs

According to the mechanism at the molecular level, chemotherapeutic drugs can be divided into four categories: alkylating agents, nucleotide reductase inhibitors and their anti-metabolites, antibiotics, and anti-tumor plant drugs [22,23]. There are dozens or even hundreds of drugs in each class. Here, the mechanism is summarized according to the active site of the chemotherapeutic drugs.

### 2.1. Destroy the Structure of DNA or Interfere with DNA Replication

Some kinds of chemotherapeutic drugs interfere with DNA replication by damaging the DNA chain, disrupting the base pair binding, or interfering with the nucleotide anabolic metabolism. Among the different types of DNA damage induced in cells, double-strand breaks (DSBs) are the most lethal if left unrepaired. Unrepaired DSBs in tumor cells exacerbate existing gene deletions, chromosome losses and rearrangements, and aberrant features that characteristically enable tumor progression, metastasis, and drug resistance. Radiotherapy, radiotherapy drugs, bifunctional alkylating agents, topoisomerase inhibitors, and replication inhibitors are all factors that can lead to DSB [24]. Alkylating agents are the earliest cytotoxic drugs and are considered to be an effective chemotherapeutic drug with a broad antitumor spectrum, short half-life in the body, and high toxicity. They are often used in high-dose short-course therapy or intermittent medication. There are many types of alkylating agents, including five categories of drugs: nitrogen mustard, ethyleneimine, nitrosourea, methyl xanthate, and epoxy compounds. Since the study of nitrogen mustard began in 1942, DNA alkylation has become an effective anticancer strategy [25]. Alkylation usually replaces a hydrogen group on a base with an alkyl group by a nucleophilic reaction (SN1 or SN2), which is usually combined with guanine (N7, O6, N2, N3), adenine (N3, N7), and cytosine (N3) equipotential binding [26], thereby preventing the expansion and binding of DNA double strands to prevent DNA replication. Although platinum drugs are also alkylating agents, they do not interact with biological macromolecules, but form a complex with the N7 position of guanine, thereby inhibiting DNA replication and transcription and inducing apoptosis [27]. DNA methylation is one of the important epigenetic mechanisms regulating cell proliferation, apoptosis, differentiation, cycle, and transformation, and plays an important role in transcriptional suppression of eukaryotes [28]. Temozolomide (TMZ) is a methylating agent approved by Food and Drug Administration (FDA) in the 1990s for the treatment of glioblastoma multiforme (GBM) and astrocytoma [29]. Being a small lipophilic molecule, TMZ penetrates the blood–brain barrier and is therefore one of the few drugs with central nervous system (CNS) activity. Prodrugs are cunning derivatives of therapeutic agents designed to improve the pharmacokinetics profile of the drug [30]. In prodrugs, the pharmacological activity of the drug is masked and restored in the human body after the biotransformation of the prodrug. TMZ as a prodrug could converted to the active metabolite 5-(3-methyltriazen-1-yl) imidazole-4-carboxamide (MTIC) by nonenzymatic chemical conversion. Among the DNA damages caused by MTIC, the most common is methylation of guanine N7, followed by methylation of guanine N3 and guanine O6. In normal cells, the MGMT enzyme directly repairs O6-MeG, which transfers the methyl group at the O6 position of guanine to the cysteine residue in the body. In cells with low MGMT levels and normal mismatch repair system (MMR), O6-MeG mismatches with thymine during replication, which is subsequently recognized by MMR, resulting in cycles of repair attempts. This can ultimately lead to strand interruptions (SSB/DSB) with a fatal cellular outcome (apoptosis) [31].

**Figure 1:**
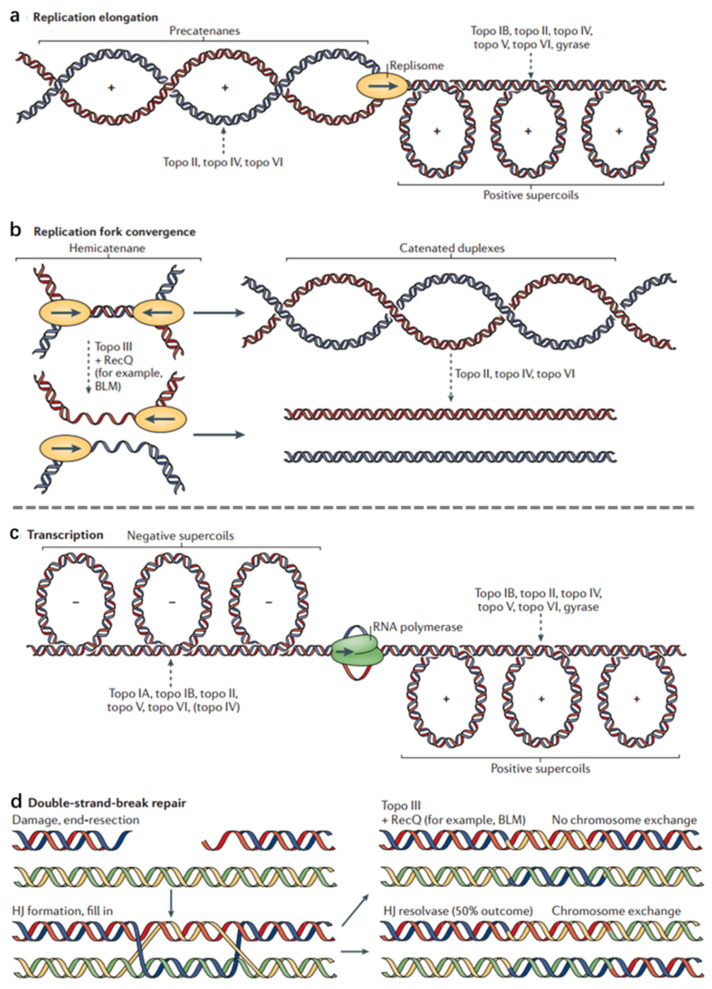
Topoisomerase functions during DNA replication and DNA repair. Topological problems that arise during DNA replication. The names of the topoisomerases that resolve these superstructures are listed. Topoisomerase action is indicated by a dashed arrow. (**a**) Replication elongation. As a replisome progresses, positive supercoils form ahead of the fork, and newly replicated precatenanes form behind it. If unresolved, precatenanes can give rise to tangled or catenated DNAs that lead to abnormal DNA segregation on entry into cell division. Unresolved positive supercoils can impede fork progression and terminate DNA replication prematurely. (**b**) Replication fork convergence. Hemicatenanes are formed as two forks converge, and must be resolved before chromosome segregation can occur. The unreplicated parental duplex can be unlinked by topo III, together with a RecQ-family helicase, after which the single-stranded gaps are filled in. Alternatively, the unreplicated parental duplex can be replicated to form a catenane with duplex linkages, which are then removed by a type II topoisomerase. (**c**) The formation of positive and negative supercoils and the names of topoisomerases acting on these superstructures. (**d**) Double-strand-break repair through homologous recombination. The broken DNA can be repaired by forming a double Holliday junction (HJ). Topo III, together with a RecQ-type helicase, can resolve these junctions, generating disentangled chromosomes that have no crossovers between DNA ends. At the same time, there is a 50% probability of producing chromosomes with chromosome arms exchanged. Adapted from [32].

Topoisomerase (Topo) is an important ribozyme in cells, and plays an important role in DNA replication, transcription, and chromatin assembly supercoils (Figure 1) [32]. Topoisomerases use active site tyrosine residues to attack the phosphodiester backbone of DNA, leading to strand breaks. Then, by rotating the second strand, the enzyme reconnects the broken DNA and releases the DNA product with a changed topology [32]. The inhibition of Topo I can cause SSBs while the inhibition of Topo II can cause DSBs [33]. Though the level of Topo II is low in cells, it is essential for cells in the proliferation and differentiation of cells entering the G2 and mitotic phases [34]. Topo I and Topo II are important targets for cancer treatment [35]. Topo I inhibitors mainly include topotecan (TPT) [36], irinotecan [37], belotecan [38], and camptothecin (CPT) [39]. CPT can block Topo I and break the DNA strand, thereby inhibiting DNA replication [40]. Irinotecan and TPT are derivatives of CPT, which can combine with Topo I and DNA to form a ternary complex [39]. In general, type I topoisomerase inhibitors can prevent the rotation of the chain or prevent the release of the reaction, thereby making the isomerase and inhibitor more firmly bound. Podophyllotoxin drugs (such as etoposide and teniposide) are type II topoisomerase inhibitors, which bind to Topo II, causing DNA strand breaks [41]. Doxorubicin (DOX) can cause single- or double-stranded DNA to break down and alter the function of nuclease to unchain the DNA from double-strand to single-strand [42]. Mitoxantrone combines with Topo II to form an easily dissociable complex, which induces DNA strand breaks. Mitoxantrone could poison Topo II, destroy the DNA structure, activate the NF-kappa B pathway, and induce apoptosis [43].

**Figure 2:**
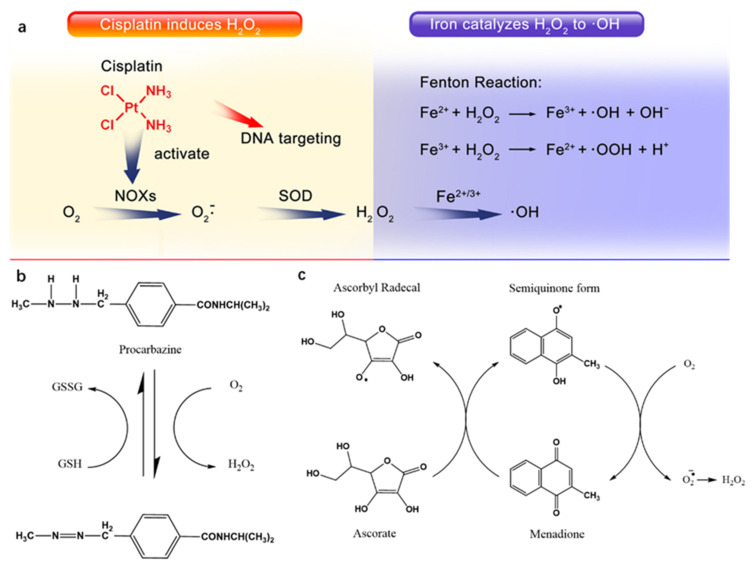
Mechanism of ROS generation by Pt, procarbazine, and menadione. (**a**) Cisplatin activates NOX, which catalyzes formation of superoxide and H_2_O_2_ from O_2_; iron catalyzes the Fenton chemistry to turn H_2_O_2_ into highly toxic •OH; adapted from [48]. (**b**) Procarbazine oxidation to its azo derivative yields hydrogen peroxide; adapted from [50]. (**c**) H_2_O_2_ is produced during the ascorbate-driven menadione redox cycling; adapted from [51].

DNA can be damaged by reactive oxygen species (ROS). ROS is a general term for a class of oxygen-containing molecules and plays an important role in cell signaling and cell stability [44,45]. A high concentration of ROS can cause DSBs and even apoptosis [46]. A large number of studies have shown that many common chemotherapeutic drugs can induce oxidative stress such as DOX, mitomycin C, mitoxantrone, carmofur, GEM, mercaptopurine, CPT, TAX, vinblastine, and vinorelbine [47]. Cisplatin (Pt), procarbazine, and quinone drugs are also common anticancer drugs that can produce ROS (Figure 2) [48,49,50]. They can produce ROS and induce DNA damage to achieve anticancer effects.

### 2.2. Inhibit Enzyme and Protein Synthesis

**Figure 3:**
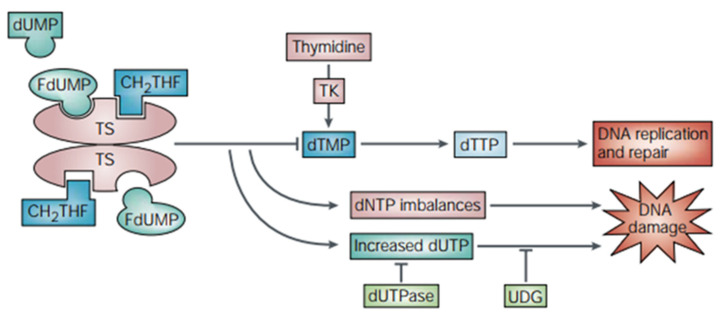
Mechanism of thymidylate synthase inhibition by 5-FU. TS catalyzes the conversion of dUMP to dTMP with 5,10-methylene tetrahydrofolate (CH2THF) as the methyl donor. The 5-FU active metabolite fluorodeoxyuridine monophosphate (FdUMP) forms a stable triple complex with TS and CH2THF, blocking access of dUMP to the nucleotide-binding site and inhibiting dTMP synthesis. This results in deoxynucleotide (dNTP) pool imbalances and increased levels of deoxyuridine triphosphate (dUTP), both of which cause DNA damage. The pyrophosphatase dUTPase and uracil-DNA glycosylase (UDG) affect the degree of DNA damage caused by dUTP. Thymidine kinase (TK) can promote the synthesis of dTMP. Adapted from [53].

The chemical structure of some chemotherapeutic drugs is similar to the necessary substances for nucleic acid metabolism. Thus, these kinds of drugs can inhibit the synthesis of cellular enzymes, hinder DNA replication, and induce cell damage and apoptosis. Thymine synthase (TS) is a folate-dependent enzyme and plays an irreplaceable role in DNA synthesis [52]. TS operates in two ways: (i) as a catalytic enzyme, TS regulates its own expression by binding and inactivating its RNA; (ii) it catalyzes the reductive methylation of 2′-deoxyuridine-5′-monophosphate (dUMP) to 2′-deoxythymidine-5′-monophosphate (dTMP) [52]. The inhibition of TS can be accomplished through the design of molecules that interfere with substrate binding. For example, FDUMP, the active form of 5-FU, can inhibit TS and induce DNA damage (Figure 3) [53]. The antimetabolite 5-FU can also inhibit deoxythymidylate synthase, prevent the synthesis of thymine nucleotides, and prevent the methylation of deoxyuracil nucleotides, thus affecting DNA synthesis [54]. Moreover, 5-FU can also bind to RNA, causing the destruction of RNA and finally being integrated into DNA, leading to DNA fragmentation [53]. In addition, drugs such as deoxyfluorouridine and pemetrexed (PTX) can also treat tumors by inhibiting TS [55].

Dihydrofolate reductase (DHFR) catalyzes the regeneration of tetrahydrofolate by reducing dihydrofolate. Tetrahydrofolate is a substance necessary for the synthesis of purine and thymine as well as glycine, methionine, and serine. DHFR inhibition disrupts the biosynthesis of purine and thymine, affects DNA replication, and leads to cell death [56]. MTX is a classic drug that inhibits DHFR, which can bind to dihydrofolate reductase so that dihydrofolate cannot be reduced to tetrahydrofolate. It can also cause 5,10-dimethyltetrahydrofolate deficiency, making deoxyuridine unacceptable for carbon units from 5,10-dimethyltetrahydrofolate to form deoxythymidylate and thus inhibiting DNA synthesis [57]. MTX also can inhibit DHFR34, which can bind to dihydrofolate reductase so that dihydrofolate cannot be reduced to tetrahydrofolate. It can also cause 5,10-dimethyltetrahydrofolate deficiency, making deoxyuridine unacceptable for carbon units from 5,10-dimethyltetrahydrofolate to form deoxythymidylate and thus inhibiting DNA synthesis. In addition, other drugs such as raltitrexed, pemetrexed, and pralatrexate can also be used as DHFR inhibitors [58].

Protein synthesis is the key to cell survival, and translation regulation is the key to post-transcriptional gene expression regulation. Disorders in this process, especially through RNA binding proteins, are associated with the development and progression of many diseases including cancer [59]. The diterpenoid nagilactone E (NLE) inhibits the proliferation of lung cancer cells by down-regulating cyclin B1-mediated G2 cell cycle arrest [60]. Actinomycin D can be inserted into the DNA double helix to form a covalent bond, destroy the DNA template function, hinder DNA replication, transcription, and translation and other functions, and interfere with rRNA transcription and protein synthesis [61]. L-asparaginase is an important enzyme capable of hydrolyzing L-asparagine to L-aspartic acid, making tumor cells synthesize protein raw material L-asparagine deficiency, and inhibiting protein synthesis in cancer cells [62].

### 2.3. Destroy Cellular Structural Components

**Figure 4:**
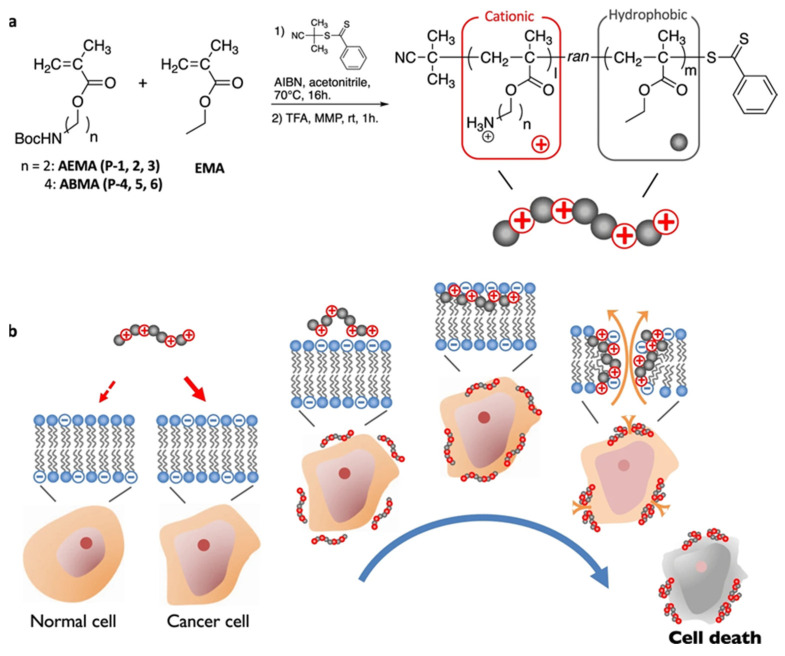
(**a**) Synthesis of methacrylate random copolymers with cationic and hydrophobic side chains. (**b**) The mechanism of prepared cationic polymers to destroy the cell membrane. Through electrostatic interaction, cationic anticancer peptides (ACPs) selectively bind to the cell membrane of PS-rich anionic cancer cells. The bound ACPs insert the hydrophobic domain of the spiral into the cell membrane, causing the cell membrane to rupture, leakage of cell components, and ultimately the death of cancer cells. Adapted from [71].

Some chemotherapeutic drugs can destroy cell structures or suborganelles, such as mitochondria, ribosomes, and Golgi apparatus, thus causing cell death. Mitochondria play an important role in cell transformation, cell biosynthesis, and energy supply, as well as the regulation of cell apoptosis and autophagy. TAX, DOX, and CPT can change the growth activity of tumor cells and induce tumor cell apoptosis by targeting mitochondria. Triphenylphosphine (TPP) is a kind of delocalized cationic lipid, which is one of the most researched targeting components in mitochondrial targeted therapy. Since the mitochondrial membrane potential is highly negative, TPP is easy to accumulate and penetrate the mitochondrial membrane [63]. Anthracyclines can quickly penetrate the mitochondria and interact with multiple molecular targets such as the multienzyme complex of electron transfer chain (ETC)/oxidative phosphorylation system (OXPHOS), mitochondrial DNA (mtDNA), and mitochondrial permeability transition pore (MPTP), leading to energy metabolism disorder and induce apoptosis [64]. Intracellular proteins are processed and secreted by the Golgi apparatus. All secreted proteins involved in tumorigenesis and development are modified, transported, and secreted by the Golgi apparatus [65]. Brefeldin A (BFA) is a signal transfer inhibitor that acts on the Golgi apparatus. After incubation, it collapses and merges into the endoplasmic reticulum, affecting vesicle transport and inhibiting the secretion of secreted proteins [66]. BFA can also increase the endoplasmic reticulum stress level of cancer cells, thereby inducing caspase-12 pathway-mediated apoptosis [67]. In addition to chemotherapeutic drugs, some polymers and nanoparticles can also destroy the cytoskeleton and morphology [68,69]. For instance, due to the overexpression of phosphatidylserine (PS) on the surface of cancer cells [70], methacrylate random copolymers can bind to PS lipids, leading to cell membrane rupture, leakage of cell components, and ultimately death of cancer cells (Figure 4) [71]. Ag nanoparticles (AgNPs) can destroy the cytoskeleton and morphological structure and change the nanostructure of the cell membrane, thereby increasing the roughness of the cell membrane, reducing the adhesion performance of the cell membrane and the stiffness of the cell [72]. At the same time, it also leads to abnormal mitochondrial function and promotes hyperpolarization of membrane potential and accumulation of ROS, thereby inducing colon cancer cell death [73].

### 2.4. Inhibit Tumor Angiogenesis

The characteristics of the infinite proliferation of tumors determine that the density of blood vessels around tumor cells is higher than that of normal cells [74]. Tumor cells secrete pro-angiogenic factors such as vascular endothelial growth factor (VEGF) to promote angiogenesis [75]. Therefore, to place tumor cells in a dormant or starvation state by inhibiting the secretion of angiogenic factors or blocking the flow of blood in the blood vessels is a promising treatment strategy for cancer [76]. TAX is a member of the taxane family and exerts an anti-tumor effect by targeting microtubules in cancer cells. Studies have shown that TAX has anti-angiogenic effects and induces tumor cell apoptosis [77]. Dihydroartemisinin (DHA), as an anti-angiogenic drug, inhibits the expression of fatty acid synthase (FASN) and inhibits endothelial cell (EC) tube production by inhibiting the STAT3 signaling pathway [78]. Bevacizumab is the first anti-angiogenic monoclonal antibody approved by the FDA [79]. Other anti-angiogenic drugs such as sunitinib, pazopanib, vandetanib, axitinib, regorafenib, cabozantinib, and lenvatinib have been approved by the FDA for the treatment of various cancer patients [80].

## 3. Cell Cycle Regulation

The cell cycle is an ordered set of events that ultimately leads to cell growth and division. The cell cycle in eukaryotic cells has traditionally been divided into two major phases: interphase and mitosis (M phase). Interphase is composed of three subphases: G1, S, and G2. In the G1 phase, the biosynthesis of RNA and protein is mainly carried out to prepare for the DNA synthesis in the S phase. During this period, the synthesis of mRNA, rRNA, and tRNA accelerates, leading to the formation of structural proteins and enzyme proteins. The S phase refers to the period from the beginning to completion of DNA replication. The most important feature of this period is the replication of DNA and the synthesis of chromosomal proteins such as histones and non-histone proteins. Through DNA replication, genetic information is accurately transmitted to the daughter cells of M phase division to ensure the stability of genetic traits. Therefore, the S phase is the most critical phase in the cell cycle. Many chemotherapeutic drugs mainly act on the S phase of cells. The G2 phase is the period from the completion of DNA replication to the beginning of mitosis. During the G2 phase, the synthesis of RNA and proteins directly related to mitosis, such as microfilaments, tubulin, and important factors in mitosis regulation, occur to prepare for mitosis. The M phase is divided into the prophase, metaphase, anaphase, and telophase, which is the process of dividing chromosomes into two daughter cells precisely and evenly. The DNA and proteins of the cell are divided equally into two cells, completing the process of cell replication. In the process of cell growth and reproduction, the end of the previous cycle is generally the beginning of the next cycle. However, some cells do not enter the next cycle but temporarily exit the cell cycle and enter the G0 phase. Cells in the G0 phase will transform to the G1 phase under the influence of mitogens. Cell cycle regulation is a series of complex mechanisms involving the regulation of a variety of cyclins, cyclin-dependent kinases, cell cycle checkpoints, and cell cycle signaling pathways.

### 3.1. Cyclin and Kinase

**Figure 5:**
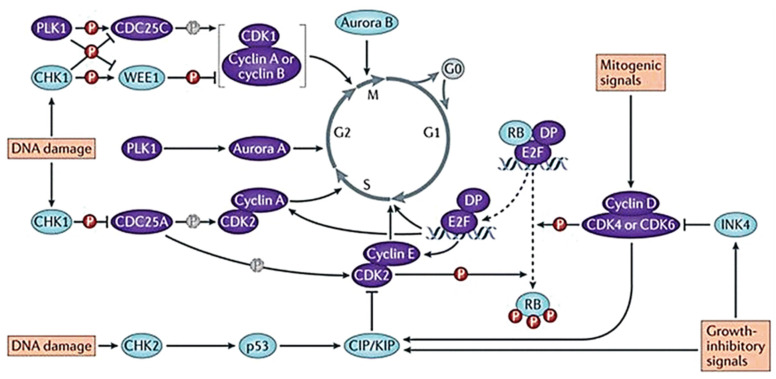
Evolution of the cell cycle and the main regulator proteins. The positive regulators of cell cycle progression are in purple while the negative regulators are in blue. Adapted from [84].

During the mitotic cycle, cell cycle regulatory proteins can bind to cycle-encoded proteins and activate corresponding protein kinases, thereby promoting cell division [81]. At least eleven different cyclins have been found, namely A, B1, B2, C, D1, D2, D3, E, F, G, and H. All kinds of cyclins contain a conserved sequence of amino acids, called cyclin frame, which mediates the binding of cyclins to CDK. In mammalian cells, CDKs, CDK inhibitors (CDKIs), and retinoblastoma proteins (Rb) strictly control the changes of cyclin in different stages. Cells lacking related cyclin are blocked at the G1/S border [82]. The activation of cyclin D and cyclin E can promote the transition of cells from G1 phase to S phase, while the activation of CDC2 can promote the transformation of cells from S phase to G2/M phase [83]. The activity of CDKs controls cell cycle transcription and plays an important role in regulating spindle polymerization checkpoints. CDKs can initiate, promote, and complete cell cycle events (Figure 5) [84]. The activation of CDKs can move the cell from the current stage to the next stage. The cell cycle is controlled by many CDK and cyclin complexes. The activated CDK1 can phosphorylate the target proteins to produce corresponding physiological effects, such as nuclear fibrin phosphorylation, leading to nuclear fibrin disintegration, nuclear membrane disappearance, and chromosome condensation. Various forms of CDC2 and CDK cyclin activation time prove that the function of CDK-G1 cyclin dimer is to regulate G1 and S phase, while CDC2-cyclin A and B regulate the mitotic process [85]. CDKIs negatively regulate the cell cycle. When cells overexpress cyclins or do not express CDKIs, cell growth will be uncontrolled. A variety of CDKIs have been discovered, such as INK4 protein and Cip/kip. INK4 is an inhibitor of CDK4 and CDK6, which can bind to CDK4/6 and interfere with the binding of these kinases to cyclin D. It can maintain the highly phosphorylated state of Rb and eventually block the cell cycle [86]. Abnormal cell cycle regulation and CDK4/6 activation are important mechanisms of tumor cell proliferation [87]. Cip/kip is not only an inhibitor of cyclin E and A-dependent kinase CDK2, but also a positive regulator of cyclin D-dependent kinase. Cip/kip causes the accumulation of cyclin D, thereby enhancing Rb phosphorylation and causing the cell to enter the S phase [88]. Rb is a tumor suppressor protein that negatively regulates cell cycle progression and is one of the CDK tumor suppressor substrates. The cyclin D–CDK4/6 complex can phosphorylate the C-terminal of Rb. Phosphorylated Rb can inhibit the binding of histone deacetylase (HDAC) by interacting with molecules in the central capsule region, thereby preventing active transcription. When Rb is not phosphorylated, it inhibits the E2F transcription factor by blocking the E2F transcription factor deactivation domain and HDAC recruitment [89]. E2F is an important regulator of cell cycle regulation. Like pocket proteins (PPs), it can regulate genes directly involved in cell processes to control different cell functions [90]. When stimulated by mitosis, cyclin D-CDK4/6 initiates phosphorylation of PPs, leading to the destruction of the E2F/PP inhibitory complex and the nuclear output of the E2F factor. At the same time, the expression of the activating factor E2F protein (E2F1, E2F2, and E2F3) stimulates the transcription of cell cycle genes, which can make the cell transition from G1 phase to S phase smoothly [90].

### 3.2. Cell Cycle Checkpoints

The cell cycle checkpoint is significant for the cell to ensure the quality of DNA replication and chromosome allocation and is the regulatory path that controls the sequence and time of cell cycle transition. When DNA is damaged, checkpoints provide repair time by blocking the cell cycle, and respond to the damage by inducing transcription or genes that promote repair [91]. Some drugs can abolish cell cycle checkpoints at critical time points in cells, so that cells with damaged DNA have no time to be repaired and enter the next stage directly, thereby activating the apoptosis pathway and causing apoptosis. The DNA damage response (DDR) is responsible for detecting DNA damage, pausing the cell cycle and initiating DNA repair. When DNA is damaged, cell cycle checkpoint can be activated in G1 phase, S phase, and at the G2/M transition. Among them, Ataxia Telangiectasia Mutated (ATM) kinase is activated by DSBs, and triggers G1 checkpoint through phosphorylation and activation of checkpoint kinase 2 (CHK2). ATM can activate CHK2, which in turn activates p53. Activated p53 can participate in many important signaling pathways that control cell proliferation and death, including cell cycle regulation, DNA repair, metabolism, senescence, autophagy, and apoptosis [92,93]. For example, p53 can directly regulate the expression levels of key kinases p21 and CDK during cell cycle progression, leading to the inhibition of cyclin E CDK2 complex and G1 blockade [94]. The p21 protein inhibits cyclin at the G1 checkpoint and affects the cell’s transition from the G1 phase to the S phase. When DNA is damaged in S phase, arising from stalled replication forks, nucleotide excision/repair process, or as intermediates of DSB resolution, the intra S phase checkpoint is activated to prevent further replication [95]. This damage is sensed by ataxia telangiectasia and rad3 associated (ATR) kinase, which induces the degradation of CDC25A protein body by activating checkpoint kinase 1 (CHK1) and blocks the further progress of S phase [96]. In addition, ATR and CHK1 can also trigger G2/M checkpoints, preventing cells with damaged DNA from entering mitosis. CHK1 also activates WEE1 through direct phosphorylation, resulting in enhanced phospho-Cdc2 (Tyr15) phosphorylation of CDK2 and CDK1 and causing cell cycle arrest in G2 phase [97]. ROS can induce CHK1 activation or directly affect the protein phosphatase Cdc25 family (Cdc25A, B, and C) to promote cell cycle arrest [98]. WEE1, a 96 kDa bispecific kinase, plays an important role in cell cycle progression by phosphorylating CDK1 at tyrosine 15, and is a key enzyme to block G2/M metastasis. WEE1 prolongs the G2 phase by controlling the activity of CDK1, so that the DDR mechanism has more time for DNA repair [94]. WEE1 is overexpressed in many tumors, such as hepatocellular carcinoma glioblastoma and melanoma [99,100,101]. In mitosis, the correct segmentation of the replicated genome is achieved through a protective mechanism called the Spindle Assembly Checkpoint (SAC), which prevents errors in chromosome separation by delaying entry into the later stages [102]. SAC inhibits ubiquitin ligase and promotes complex/cyclic body (APC/C) in the later stage, delaying the degradation of cyclin B and the later inhibitor securin until all chromosomes are bipolar connected. SAC is applied by recruiting unattached or tension-free centromeres to the mitotic checkpoint complex (MCC) [95].

### 3.3. Cell Cycle Signaling Pathways

The cell cycle can be regulated by a variety of signaling pathways, such as ATR-CHK1/ATM-CHK2, JAK-STAT signaling pathway, p53 signaling pathway, NF-κB signaling pathway, or PI3/AKT/mTOR signaling pathway [94,103,104,105,106]. By affecting the expression of genes, proteins, or kinases in these signaling pathways, the cell cycle can be regulated [107,108].

**Figure 6:**
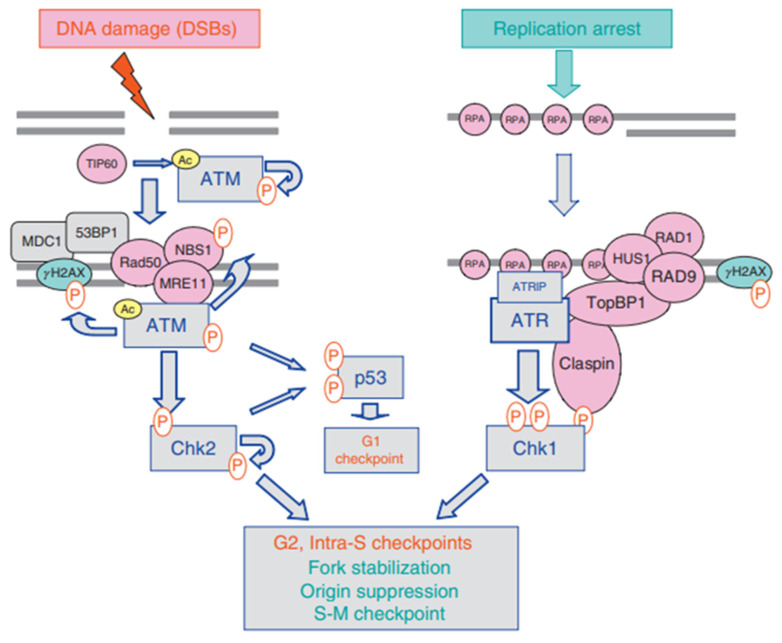
Activation of the ATM-Chk2 and ATR-Chk1 pathways. The ATM-Chk2 and ATR-Chk1 pathways are activated selectively by DSBs and tracts of ssDNA complexed with RPA, respectively. Phosphorylation events are indicated by (P) in red and acetylation by (Ac) in yellow. Adapted from [111].

ATR-CHK1 and ATM-CHK2 are two different signal cascades of DDR, activated by DSBs and SSBs, respectively. The activation of these pathways is essential for the coordination of checkpoints and DNA repair processes. In undamaged cells, ATM is thought to exist as an inactive homodimer. In response to DSBs, the inactive ATM homodimer was rapidly induced into intermolecular autophosphorylation, resulting in dissociation to form part of the active monomer [109]. When ATM is activated, it will induce the activation of CHK2 and the conduction of downstream signaling pathways. ATR is activated by many types of DNA damage, including DSB, base adducts, cross-linking, and replication stress [110]. The activation of ATR-CHK1 and ATM-CHK2 involves the phosphorylation of quantities proteins (Figure 6) [111]. The association of ATR with single-stranded DNA (ssDNA) coated with Replica Protein A (RPA) is necessary to activate ATR [112]. The activation of ATR will cause the up-regulation of p21CIP1, and then CHK1 will be activated. After activation of the ATR-CHK1 and ATM-CHK2 pathways, cell cycle checkpoints are initiated, cells enter the DNA repair phase, and the cell cycle is blocked [113]. Activation of these two pathways blocks the S, G1, and G2 phases of DNA-damaged cells [111]. Correspondingly, inhibiting the ATR-CHK1 and ATM-CHK2 pathways will inhibit cell cycle checkpoints, allowing cells with damaged DNA to enter the mitotic cycle directly without going through the repair phase, and promote cell cycle progression [111].

JAK-STAT3 signal is known for its role in tumor cell proliferation, survival, invasion, and immunosuppression [114]. The Janus kinase (JAK)-signal transducer of activators of transcription (STAT) pathway is regulated at multiple levels. JAKs can be negatively regulated by inhibiting cytokine signaling proteins (SOCS), protein tyrosine phosphatases (PTPs), etc. [115]. Intracellular PTPs (such as PTP1B and TCPTP), PIAS protein, and nuclear PTPs (such as TCPTP and SHP2) can all negatively regulate STATs. Activated STATs inhibitors interact with STATs under the stimulation of cytokines and inhibit the transcriptional activity of STATs through different mechanisms [116]. The p53 protein is the product of mutations in the p53 gene. It plays an anti-proliferative effect in different types of stress responses, including cell cycle arrest and apoptosis. For example, when cells are damaged or cell proliferation is abnormal, the p53 gene is activated, leading to cell cycle arrest and even cell apoptosis [117]. In tumor cells, p53 is easily affected by mutated genes and cellular proteins. Mdm2 oncoprotein is a potent inhibitor of p53. Mdm2 binds to the transcriptional activation domain of p53, blocking its ability to regulate target genes and exerting anti-proliferation effects. On the other hand, p53 activates the expression of Mdm2 gene in a self-regulating feedback loop [118]. The use of Mdm2 small molecule antagonists such as Nutlin-1 can activate the p53 pathway, cause cell G1 and G2 phase arrest, and inhibit tumor growth [119]. NF-κB (nuclear factor-kappa B) is one of the members of the Rel family of eukaryotic transcription factors, and it is widely found in mammalian cells. The NF-κB signaling pathway is a multi-component pathway that regulates the expression of hundreds of genes. These genes participate in various key processes of cells and organisms, including cell proliferation, cell survival, cell stress response, innate immunity, and immunity inhibition [120]. In tumor cells, NF-κB is over-expressed than normal cells, and it has a significant promotion effect on tumor metastasis [121]. The PI3/AKT/mTOR signaling pathway is important in regulating signal transduction, cell proliferation, apoptosis, metabolism, angiogenesis, and other biological processes [122]. PI3/AKT/mTOR signaling pathway is a very potential signaling pathway in tumor therapy. PI3/AKT/mTOR participates in the cell cycle process in cancer cells and promotes the occurrence and development of tumors [123]. At the same time, studies have shown that targeting PI3K/AKT/mTOR-mediated autophagy can inhibit tumor growth [122].

## 4. Advances in the Combination of Cell Cycle Regulation and Chemotherapy for Cancer Therapy

### 4.1. Strategies to Inhibit the Cell Cycle

DDR can suspend the cell cycle in conjunction with cell cycle checkpoints after DNA damage and initiate DNA repair. ATR and PARP are DNA repair proteins that transmit signals to S and G2/M phase checkpoints under the stimulation of replication stress (RS), blocking the cell cycle for DNA repair. Thus, ATR and PARP inhibitors would interfere with DNA repair [124]. Some typical cell cycle targeting reagents are summarized in Table 1. They have been used in combination with chemotherapeutic drugs, especially alkylating agents and other DNA-damaging chemotherapeutic drugs, and ideal therapeutic effects were shown [125]. Withaferin A (WA) can inhibit ATR and ATR’s downstream kinase CHK1, thus blocking the G2/M phase of cells [126]. WA combined with Pt can reduce the resistance and toxicity during chemotherapy, and good efficacy in the treatment of 3D-cultured breast cancer cells was observed [127]. nLs-BG129 is a nano-liposome ATR inhibitor with good stability and long clearance time in vivo. It can enhance the antitumor activity of chemotherapeutic drugs such as GEM and carboplatin in vivo [128]. Diallyl disulfide (DADS) is a natural organic sulfide that acts as a DNA repair inhibitor by inhibiting the protein levels of the DNA resection-related proteins Sae2 and Exo1. It can stimulate cell cycle arrest and promote apoptosis as well as prevent invasion and angiogenesis [129]. M6620, M4344, AZD6738, and BAY1895344 are all ATR inhibitors currently in clinical development. It has been proven in clinical trials that the combination of these ATR inhibitors and platinum-based chemotherapeutic drugs (e.g., Pt, carboplatin, oxaliplatin), antimetabolite-based chemotherapeutic drugs (e.g., GEM), and topoisomerase inhibitors (e.g., CPT, irinotecan, TPT) can effectively increase the sensitivity of cancer cells to chemotherapeutic drugs and, meanwhile, reduce the resistance of cancer cells to chemotherapeutic drugs [130]. The combination of the ATR inhibitor VE-822 and oxaliplatin produced a strong synergy in six different colorectal cancer cell lines and their oxaliplatin-resistant subclones. This promotes the formation of SSBs and DSBs, growth arrest, and apoptosis [131]. In addition, the synergistic effect of VE-822 with the TOP1 inhibitor irinotecan increases the sensitivity of SLFN11-negative PDXs in TNBC to irinotecan and eliminates irinotecan-induced CHK1 phosphorylation [37]. Histone methylation regulates chromatin structure and participates in DNA repair. Chemical inhibitors of JMJD2 family proteins increase H3K9me3 and H3K36me3 levels, reduce the chromatin association of ATR and CHK1, inhibit ATR-CHK1 replication checkpoints, and increase the sensitivity to cisplatin-resistant cells [132].

**Figure 7:**
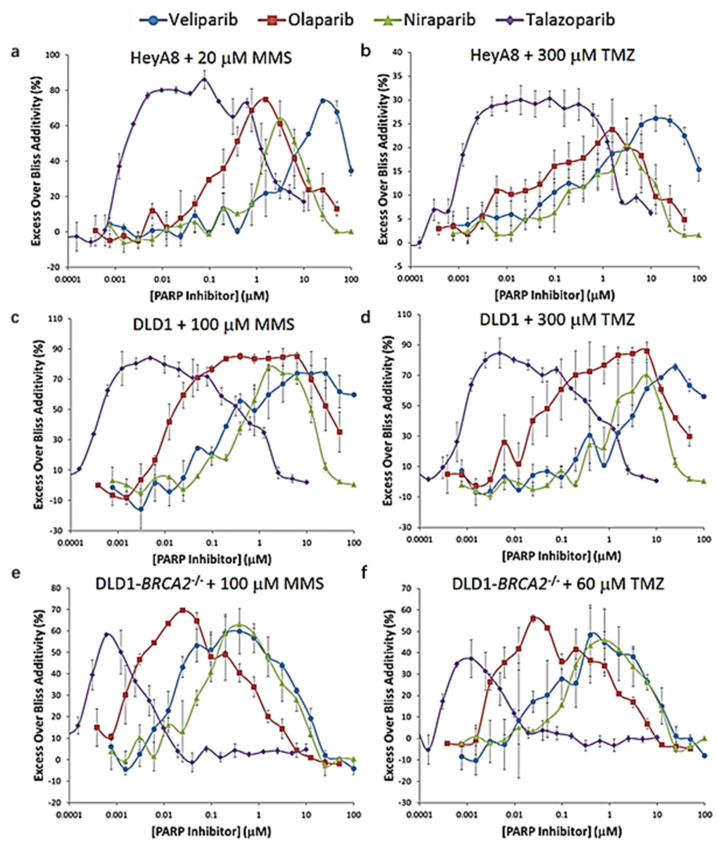
The synergistic effect of PARP inhibitor and DNA alkylating agent methyl methanesul-fonate (MMS) or TMZ in vitro. Cells were treated with two-dimensional dose responses of PARP inhib-itors and DNA alkylating agents for 5 days. Excess over Bliss additivity was determined for each condition. To facilitate comparison of PARP inhibitors, cross-sections of response surfaces at the concentrations of alkylating agent eliciting peak synergism are overlaid. In this analysis, values of zero indicate no activity or additivity whereas higher values indicate stronger synergism. The decreases observed at higher PARP inhibitor concentrations are due to a loss of synergism to single-agent PARP inhibitor activity. (**a**) HeyA8 cells with 20 μmol/L MMS. (**b**) HeyA8 cells with 300 μmol/L TMZ. (**c**) DLD1 cells with 100 μmol/L MMS. (**d**) DLD1 cells with 300 μmol/L TMZ. (**e**) DLD1-*BRCA2*^-/-^ cells with 20 μmol/L MMS. (**f**) DLD1-*BRCA2*^-/-^ cells with 60 μmol/L TMZ. Data represent means with standard errors from at least two independent experiments run in dupli-cate. Adapted from [125].

PARP is a key regulator of DDR and replication fork stability [133]. Olaparib, niraparib, velaparib, rucaparib, and talazoparib are common PARP inhibitors [134]. PARP inhibitors and alkylating agents can produce synergistic effects in cancer treatment (Figure 7) [125]. Inhibition of PARP activity leads to the capture of PARP-1 at the site of DNA damage, further inhibiting the recruitment of stagnant replication forks by HR repair enzymes [135]. Busulfan can slow down and prevent replication forks, and is indispensable in the role of conditions before transplantation. Combined treatment of PARP-1 inhibitor veliparib and alkylating agents (such as busulfan and melphalan) in vivo can lead to synergistic cytotoxicity of high-risk myeloproliferative tumor (MPN) cells [136]. In addition, in clinical trials, PARP inhibitors have been used in combination with cytotoxic chemotherapy that requires PARP-1 activity for DNA repair, including alkylating agents, Topo I inhibitors, and platinum drugs [137]. PARP inhibitors have been extensively studied in combination with chemotherapeutic drugs in cancers such as ovarian cancer, breast cancer, small cell lung carcinoma (SCLC), prostate, and pancreatic cancer [138,139,140,141].

Prexasertib (LY2606368) is an ATP competitive inhibitor of CHK1 and CHK2. LY2606368 alone can induce DNA damage and tumor cell apoptosis. Preclinical data proved that the use of LY2606368 in combination with other drugs (antimetabolites, PARP inhibitors, and platinum-based chemotherapy) in solid tumors had preferable efficacy [84]. AZD7762 is also a CHK1 inhibitor which can induce p53-deficeint, partially activate caspase 2 and downregulate E2F1 in combination with Pt, enhance the anti-tumor activity of Pt in SCLC, and overcome Pt resistance [142].

THZ531 is a potent inhibitor of CDK12. The inhibition of CDK12 can hinder the cell cycle progression. In the treatment of anaplastic thyroid carcinoma (ATC), THZ531 inhibits CDK12 and help overcome the resistance of ATC to adriamycin and other conventional chemotherapeutic drugs [143]. Dinaciclib is a small molecule inhibitor of CDK1, CDK2, CDK5 and CDK9. With the increase in intracellular ROS levels, dinaciclib alone can induce cell growth inhibition, cell cycle arrest, and apoptosis, while related proteins such as CDKs, cyclins, Mcl-1, XIAP, and survivin all change significantly. Dinaciclib combined with Pt synergistically promotes cell cycle arrest and apoptosis and inhibits the growth of nude mouse ovarian cancer subcutaneous xenograft tumors [144]. Src is a non-receptor tyrosine kinase that participates in the crossover and mediation of many signaling pathways, promoting cell proliferation, adhesion, invasion, migration, and tumorigenesis [145]. Saracatinib is a Src/Abl kinase inhibitor. Sacatinib combined with 5-FU or Pt has a synergistic effect on saracatinib-sensitive cells and saracatinib-resistant cells, which is an effective strategy for the treatment of gastric cancer [146,147].

Polo-like kinase 1 (PLk-1) is a serine-threonine kinase that is involved in mitosis and does not depend on cyclin [148]. In mammalian cells, PLk-1 is mainly located in the centrosome, responsible for centrosome separation and maturation. Studies have shown that the reduction of PLk-1 expression mediated by siRNA leads to inhibition of G2/M phase of the cell cycle [149,150]. When exposed to GEM, the cell viability and survival rate were significantly reduced, effectively inhibiting the malignant growth of pancreatic tumor cells [151].

As a therapeutic agent and dietary supplement, silibinin is well tolerated and basically has no adverse side effects on the human body [152]. DOX is one of the commonly used chemotherapeutic drugs in the clinic and belongs to anthracycline anticancer drugs. DOX can be inserted into DNA and destroy Topo II used for DNA repair, finally stopping the DNA replication process and preventing DNA from being re-encapsulated [153]. Silibinin synergistic DOX in cell cycle progression can cause G2/M arrest. Silibinin combined with DOX can produce three times the effect of using two drugs alone to treat cancer [154]. At the same time, silibinin can prevent the toxic and side effects of DOX on the body and reduce the body’s resistance to DOX [155].

B7-H3 as an immune checkpoint can affect the sensitivity of several cancer types including colorectal cancer (CRC) to various anticancer drugs and targeted therapies [156]. The overexpression of B7-H3 promotes colony formation and cell viability of CRC cells, and significantly reduces apoptosis caused by chemotherapy. With the stable down-regulation of B7-H3, the sensitivity of CRC cells increased significantly. Some preliminary evidence suggests that B7-H3 can modulate the DNA repair mechanism or drug resistance of tumor cells affected by stem cells [157]. B7-H3 can increase resistance to chemotherapeutic drugs (oxaliplatin or 5-FU) by reducing CDC25A-dependent G2/M phase blockade [158]. B7-H3 regulates the expression of CDC25A in CRC cells through the STAT3 signaling pathway. Silencing CDC25A or treating with CDC25A inhibitors can reverse B7-H3-induced cancer cell resistance [158]. In addition, compared with normal adjacent tissues, B7-H3 and CDC25A in CRC samples were significantly up-regulated and were related to the tumor stage. CDC25A is positively correlated with B7-H3 expression. CRC cells provide an alternative mechanism for obtaining chemotherapy resistance through the B7-H3/CDC25A axis [158]. B7-H3 can promote the progression of T cell lymphoma, while the silencing of B7-H3 can enhance the sensitivity of Maver and Z138 cells to rituximab and bendamustine [159]. Down-regulation of B7-H3 significantly reduced the growth and colony-forming ability of acute monocytic leukemia U937 cells, and significantly enhanced the sensitivity of U937 cells to first-line chemotherapeutic drugs (arrubicin and cytarabine) [160]. The silence of B7-H3 increases the sensitivity of human pancreatic cancer cell line Patu8988 to GEM [161]. In addition, the silencing of B7-H3 increases the sensitivity of multiple human breast cancer cell lines to TAX by phosphorylating JAK2/STAT3 to regulate the G1/G0 phase of cells [162]. Ectopic expression of B7-H3 diminished the sensitization role of astragaloside IV in cellular responses to Pt in NSCLC cells [163]. B7-H3 can decrease oxaliplatin-induced DNA damage by promoting the expression of XRCC1 via the PI3K/AKT signaling pathway [164].

Bortezomib (BTZ) is a small molecule proteasome inhibitor that mainly inhibits 26S proteasome, thereby inhibiting many processes involved in cell cycle regulation, apoptosis, cell adhesion, angiogenesis, and chemical resistance [165]. Studies have shown that BTZ can increase the sensitivity of cancer cells to chemotherapeutic drugs and effectively reduce the resistance of cancer cells to chemotherapy [166]. BTZ has a significant cytotoxic effect on NSCLC. It can induce NSCLC cell concentration and time-dependent G2/M phase arrest, and can also induce apoptosis of Bcl-2 overexpressing cells. BTZ combined with GEM or carboplatin has a significant effect in the treatment of advanced NSCLC [167]. BTZ combined with TAX or carboplatin simultaneous radiotherapy has also proved to be a good treatment for stage III NSCLC [168]. For the therapy of NSCLC, BTZ can also be combined with drugs such as docetaxel, pemetrexed, bevacizumab, vorrestat, and erlotinib [169]. BTZ combined with chemotherapeutic drugs also has a good anticancer effect in the treatment of other cancers. Lenalidomide-BTZ-dexamethasone proved to be an effective treatment in the treatment of multiple myeloma [170]. BTZ plus melphalan-prednisone and BTZ with thalidomide plus dexamethasone are also effective treatments for multiple myeloma [171,172].

### 4.2. Strategies to Activate the Cell Cycle

There are many reasons for tumor resistance to chemotherapy. Among them is the reduction in hormones during chemotherapy inducing a G0/G1 blockade of the tumor, leaving the cancer cells in a low metabolic state, which may be the main reason for chemoresistance [173]. Hormones can promote the proliferation of G0 tumor cells and make the cells enter the mitotic cycle. For example, gonadal hormone, growth hormone, thyroid hormone, insulin, etc., can increase the proportion of tumor cells in S phase and play a role in chemosensitizing of the tumor [174]. In the mouse experiment, the survival time of mice injected with growth hormone and carboplatin was significantly longer than that of mice injected with growth hormone and carboplatin alone as well as somatostatin plus carboplatin [175]. Recombinant human granulocyte-macrophage colony stimulating factor (rhGM-CSF) can shorten the time of neutropenia after chemotherapy and reduce the incidence of neutropenia-related infections and hospital stays. In vitro and in vivo, GM-CSF can transfer the cell cycle of myeloid leukemia cells from G0 phase to S phase, thereby increasing the sensitivity to cell cycle-dependent cytostatics. Thus, GM-CSF can be used as an adjuvant therapy for chemotherapy [176]. In addition, some studies have proposed ways to increase the sensitivity of chemotherapy and reduce the chance of tumor recurrence by activating dormant cancer stem cell (CSC) to enter the cell cycle [177].

When DNA is damaged, ATM and ATR are activated, thereby starting checkpoint control, stopping the cell cycle process, and coordinating the repair of DNA damage. When the activity of CHK1 or CHK2 is inhibited, the cell cycle checkpoint will be abolished, thereby promoting the cell cycle process with unrepaired DNA and ultimately leading to cell apoptosis [178]. The combination of checkpoint kinase inhibitors and DNA-damaging chemotherapeutic drugs can effectively kill tumor cells. For example, UCN-01 is an indocarbazole ATP analog, which can inhibit CHK1 and increase the sensitivity of tumor cells to the antiproliferative effects of Pt, CPT, and DOX [179]. LY2606368 has shown a good cancer cell killing rate in combination with antimetabolites or platinum-based chemotherapeutic drugs in clinical trials [84]. For example, LY2606368 combined with Pt, cytarabine, etoposide, fludarabine, GEM, mitoxantrone, olaparib, lametinib, and other drugs showed good efficacy [180,181]. Polypurine Reverse Hoogsteen hairpins (PPRHs) are used as inhibitors of CHK1 and WEE1 in combination with DNA damage drugs such as MTX and 5-FU, showing a synergistic effect [182]. Adavosertib (AZD1775) is an inhibitor of WEE1 kinase, which can enhance the replication stress caused by oncogenes or chemotherapy. It is used in combination with irinotecan in relapsed or refractory solid tumors and primary central nervous system tumors [183]. AZD1775 is a WEE1 inhibitor that causes a significant increase in γH2AX levels in TNBC cells, S phase arrest, and caspase-mediated cell death [184]. The combination therapy of AZD6738 and AZD1775 activates CDK1 activity to force cells to enter mitosis and cause DNA damage. This process can induce severe mitotic abnormalities and mitotic disasters and, furthermore, also make TNBC cells sensitive to Pt and PARP inhibitors [185]. SRA737 is a CHK1 inhibitor used in combination with low-dose GEM to produce an anti-tumor response in various cancer models, including SCLC [186]. XL-844 is an effective inhibitor of CHK2. XL-844 and GEM are used in clinical trials to treat advanced solid tumors and lymphomas [187]. In GEM- and XL-844-treated PANC-1 cells, the S-phase checkpoint was overcovered. This resulted in phosphorylation of CHK1 and increased levels of H2AX. The cell then enters mitosis prematurely and reduces the survival rate of clone formation [188]. GDC-0575 is a highly selective oral small molecule CHK1 inhibitor, which can cause tumor contraction and growth delay in xenograft models [189]. It can be used as a single agent or in combination with GEM. In clinical trials, GDC-0575 combined with GEM proved to be a safe and gentle method for treating various solid tumors [190]. PF-00477736, LY2606318, MK-8776, GDC-0425, GDC-0575, SRA737, and other drugs are all clinically developed CHK1 inhibitors, which can be used in combination with Pt or GEM and other chemotherapeutic drugs [191].

## 5. Conclusions and Perspectives

Most chemotherapeutics act on cells in the proliferative phase. Therefore, reasonable artificial regulation of the cell cycle will have a positive impact on chemotherapy. Clinically, for solid tumors, surgery or radiotherapy is usually used to reduce the tumor volume first, and then remove the remaining cancer cells through chemotherapy. Surgery or radiotherapy will stimulate dormant tumor cells to re-enter the replication stage, and the cell cycle-specific chemotherapy drugs that follow will wipe them out. It is meaningful to further develop new coordination strategies while simplifying the treatment steps while improving the effect. The synergistic mechanism and potential antagonism need to be studied in depth. Although many combined strategies have achieved good results in animal experiments, it is a long way from clinical application. Nano-drug delivery systems (NDDS) can help drugs overcome the delivery barriers, reduce the side effects of chemotherapy, and achieve precise treatment. The proposed NDDS may make cell cycle regulation strategies more effective in combination with chemotherapeutic drugs.

## Figures and Tables

**Table 1:** Cell cycle targeting reagents.

Target Cell Cycle	Target	Reagent	Ref.
G2/M	CHK1	VE-822	[37]
	CHK1, CHK2	Prexasertib (LY2606368)	[84]
	ATR, CHK1	Withaferin A	[126]
	ATR	nLs-BG129	[128]
	ATR	M6620	[130]
	ATR	M4344	[130]
	ATR	AZD6738	[130]
	CHK1	AZD7762	[142]
	CHK1	Silibinin	[152]
		Bortezomib	[167]
		SRA737	[186]
	WEE1	Adavosertib (AZD1775)	[183]
G0/S		rhGM-CSF	[176]

## Data Availability

Not applicable.

## Abbreviations

MDR	Multidrug resistance
GEM	Gemcitabine
TAX	Paclitaxel
DOX	Doxorubicin
ROS	Reactive oxygen species
CCSA	Cell cycle specific agents
CCNSA	Cell cycle nonspecific agents
HSCLC	Human non-small cell lung cancer
CNS	Central nervous system
TMZ	Temozolomide
DDS	Drug delivery systems
Topo	Topoisomerase
DSB	Double-strand breaks
TPT	Topotecan
CPT	Camptothecin
TS	Thymine synthase
5-FU	5-fluorouracil
PTX	Pemetrexed
NLE	Nagilactone E
TPP	Triphenyl phosphonium
ETC	Electron transfer chain
OXPHOS	Oxidative phosphorylation system
MtDNA	mitochondrial DNA
MPTP	Mitochondrial permeability transition pore
DHAQ	Mitoxantrone
ROSC	Roscovitine
CLX	Celecoxib
BFA	Brefeldin A
PS	Phosphatidylserine
